# Dissemination and Characterization of Plasmids Carrying *oqxAB*-*bla*_CTX-M_ Genes in *Escherichia coli* Isolates from Food-Producing Animals

**DOI:** 10.1371/journal.pone.0073947

**Published:** 2013-09-09

**Authors:** Bao-Tao Liu, Qiu-E Yang, Liang Li, Jian Sun, Xiao-Ping Liao, Liang-Xing Fang, Shou-Shen Yang, Hui Deng, Ya-Hong Liu

**Affiliations:** College of Veterinary Medicine, National Reference Laboratory of Veterinary Drug Residues (SCAU), South China Agricultural University, Guangzhou, Guangdong, China; Institut National de la Recherche Agronomique, France

## Abstract

**Background:**

The association of PMQR and ESBLs in negative-bacteria isolates has been of great concern. The present study was performed to investigate the prevalence of co-transferability of *oqxAB* and *bla*_CTX-M_ genes among the 696 *Escherichia coli* (*E. coli*) isolates from food-producing animals in South China, and to characterize these plasmids.

**Methods:**

The ESBL-encoding genes (*bla*_CTX-M_, *bla*_TEM_ and *bla*_SHV_), and PMQR (*qnrA*, *qnrB*, *qnrS*, *qnrC*, *qnrD*, *aac*(*6*’)*-Ib-cr*, *qepA*, and *oqxAB*) of these 696 isolates were determined by PCR and sequenced directionally. Conjugation, S1 nuclease pulsed-field gel electrophoresis (PFGE) and Southern blotting experiments were performed to investigate the co-transferability and location of *oqxAB* and *bla*_CTX-M_. The *EcoR*I digestion profiles of the plasmids with *oqxAB-bla*_CTX-M_ were also analyzed. The clonal relatedness was investigated by PFGE.

**Results:**

Of the 696 isolates, 429 harbored at least one PMQR gene, with *oqxAB* (328) being the most common type; 191 carried *bla*_CTX-M_, with *bla*_CTX-M-14_ the most common. We observed a significant higher prevalence of *bla*_CTX-M_ among the *oqxAB*-positive isolates (38.7%) than that (17.4%) in the *oqxAB*-negative isolates. Co-transferability of *oqxAB* and *bla*_CTX-M_ was found in 18 of the 127 isolates carrying *oqxAB*-*bla*_CTX-M_. These two genes were located on the same plasmid in all the 18 isolates, with *floR* being on these plasmids in 13 isolates. The co-dissemination of these genes was mainly mediated by F33:A-: B- and HI2 plasmids with highly similar *EcoR*I digestion profiles. Diverse PFGE patterns indicated the high prevalence of *oqxAB* was not caused by clonal dissemination.

**Conclusion:**

*bla*_CTX-M_ was highly prevalent among the *oqxAB*-positive isolates. The co-dissemination of *oqxAB*-*bla*_CTX-M_ genes in *E. coli* isolates from food-producing animals is mediated mainly by similar F33:A-: B- and HI2 plasmids. This is the first report of the co-existence of *oqxAB*, *bla*_CTX-M_, and *floR* on the same plasmids in *E. coli*.

## Introduction

Quinolone resistance was thought to be mediated only by chromosomal mutations, until plasmid-mediated quinolone resistance (PMQR) was described in 1998 [1]. Since then, a number of plasmid-mediated quinolone resistance (PMQR) mechanisms have been described: the pentapeptide repeat family Qnr proteins (QnrA, QnrB, QnrS, QnrC, and QnrD) [1,2,3,4,5], AAC(6’)-Ib-cr, an aminoglycoside acetyl- transferase that is responsible for reduced susceptibility to ciproﬂoxacin by modifying ciproﬂoxacin [6], QepA, an efﬂux pump belonging to the major facilitator subfamily [7], and OqxAB, a multidrug efﬂux pump that confers resistance to multiple agents, which has been recently reported to reduce susceptibility to ciproﬂoxacin and nalidixic acid [8]. The PMQR genes confer only low-level resistance to quinolones; however, they can be spread horizontally among enterobacteria and facilitate the selection of resistant mutants following exposure to ciprofloxacin [9]. Fluoroquinolones, and cephalosporins are commonly used to treat gram-negative bacterial infections, especially some intestinal or extraintestinal infections caused by *E. coli*. Increasing resistant isolates, especially multidrug-resistant *E. coli* isolates, have been observed [10,11], due to the use of these antimicrobials, both in human and animal diseases over the past decades.

The presence of multidrug-resistant isolates harboring multiple resistance genes on the same plasmid has been of great concern, as it expands the subset of drugs that may select for the dissemination of multidrug resistance plasmids and poses a serious risk to both animal and human health. Except for *oqxAB*, other PMQR genes were often found to be strongly associated with extended-spectrum β-lactamase (ESBL) genes, and some were often found to be located on the same plasmid [9]. Since *oqxAB* was reported to be related to reduced susceptibility to ciproﬂoxacin and nalidixic acid [8], it has been found among *E. coli* isolates from animals and humans [12,13,14]. Reports on the prevalence of coexistence of PMQR (including *oqxAB*) and ESBL genes in the same isolate have increased in the past years [12,15]. However, there is a paucity of data with regard to the prevalence and characterization of plasmids co-carrying *oqxAB*-*bla*_CTX-M_ genes in bacteria, except only one *E. coli* isolate in our previous report [16]. Because antibiotic resistant bacteria from food-producing animals can be transferred to humans through the food chain or other routes [17,18], monitoring antimicrobial resistance in bacteria from the food-producing animals is important for ensuring food safety and public health.

The present study was conducted to investigate the clinical *E. coli* isolates from food-producing animals in China for the prevalence and dissemination of plasmids harboring *oqxAB*-*bla*_CTX-M_ genes, but also the characterization of these plasmids.

## Materials and Methods

### Bacterial isolates

A total of 696 non-duplicate *E. coli* isolates (318 avian including 177 from ducks, 110 from chickens and 31 from geese, and 378 from pigs) were isolated from diseased food-producing animals between March 2002 and August 2012. The animals were from more than 80 farms all over Guangdong province. Animals we chose liver or heart tissues to obtain isolates were infected with *E. coli*, and the other animals showed diarrhea. Further information about these animals, the underlying disease and possible antimicrobial pretreatment were unfortunately not available. Cotton swabs of the liver and heart tissues or faeces from these animals were streaked onto MacConkey agar. After 16h incubation at 37°C, one colony with typical *E. coli* morphology was selected and purified on MacConkey agar. One colony was selected from each sample and all *E. coli* isolates were identified by classical biochemical methods and confirmed using the API 20E system (bioMe ´rieux). All identified isolates were stored at -80°C in Luria–Bertani broth containing 30% glycerol. *E. coli* C600, resistant to streptomycin, was used as the recipient strain in the conjugation experiments.

### Antimicrobial susceptibility testing

Susceptibilities to enrofloxacin, ciproﬂoxacin, levoﬂoxacin, nalidixic acid, amikacin, gentamicin, ﬂorfenicol, chloramphenicol, ampicillin, ceftiofur, cefotaxime, doxycycline, and tetracycline of the 696 isolates were assayed by the agar dilution method, according to the guidelines of the Clinical and Laboratory Standards Institute (CLSI) [19]. *Escherichia coli* ATCC 25922 was used as the control strain. Isolates were classiﬁed as either susceptible or resistant according to the interpretative standards recommended by the CLSI [19]. As there are no CLSI breakpoints for ﬂorfenicol and ceftiofur that are applicable to *E. coli* of animal origin, the breakpoints of ﬂorfenicol (≥16mgL^-1^) and ceftiofur (≥ 8mgL^-1^) were sourced from the Danish Integrated Antimicrobial Resistance Monitoring and Research Program [20] and a previous report [21]. ESBL-producing isolates were screened by double-disk synergy test using both cefotaxime and ceftazidime in the presence or absence of clavulanic acid as recommended by the CLSI.

### Detection of PMQR determinants

PMQR genes including *qnrA*, *qnrB*, *qnrS*, *qnrC*, *qnrD*, *aac(6*’)*-Ib-cr*, *qepA*, *oqxA*, and *oqxB* among the 696 clinical *E. coli* isolates were analyzed by PCR ampliﬁcation using previously described primers [2,5,16,21,22]. The association of IS26 with *oqxAB* was investigated by PCR as reported previously [23], using forward primer IS26-F (5’-GCTGTTACGACGGGAGGAG-3’) and reverse primer *oqxA*-R (5’-GAGGTTTTGATAGTGGAGGTAGG-3’). The DNA sequences obtained after direct sequencing of the ampliﬁcation products were conﬁrmed using the BLAST algorithm available through the National Center for Biotechnology Information (NCBI).

### Detection of ESBL-encoding genes

ESBL-encoding genes (*bla*_TEM_, *bla*_SHV_, *bla*_CTX-M-1G_, *bla*_CTX-M-9G_, *bla*_CTX-M-2G_, and *bla*_CTX-M-25G_) among the ESBL-producing isolates were analyzed by PCR ampliﬁcation using previously published primers and protocols [24,25,26]. Among the 136 isolates harboring *bla*_CTX-M-9G_, 89 were selected randomly to be directly sequenced using the PCR products, and 33 out of the 63 *bla*_CTX-M-1G_-positive isolates were randomly selected to be sequenced. The DNA sequences obtained were compared with genes in GeneBank (http://www.ncbi.nlm.nih.gov/) to conﬁrm the subtypes of ESBL-encoding genes.

### Conjugation experiment

Isolates harboring *oqxAB* and genes encoding ESBLs, were selected for conjugation experiments by the broth-mating method using *E. coli* C600 as the recipient [27]. Transconjugants were selected on MacConkey agar plates containing streptomycin (1,000mg/L) and cefotaxime (2mg/L). The transconjugants harboring *oqxAB* and ESBL-encoding genes mentioned above were conﬁrmed by PCR as previously and antimicrobial susceptibility testing for the transconjugants, recipient, and donors.

### Plasmids analysis of transconjugants

Incompatibility (Inc) groups were assigned by PCR-based replicon typing of transconjugants [28]. To better clarify IncF plasmids, replicon sequence typing of IncF plasmids was carried out according to the method reported previously [29]. Alleles were assigned by submitting the amplicon sequence to the plasmid multilocus sequence typing (pMLST) database (http://www.pubmlst.org/plasmid).

To analyze the location of the *oqxAB* gene and ESBL-encoding genes of transconjugants, S1 nuclease-PFGE and Southern blot analysis were performed. Briefly, whole-cell DNA of the transconjugants co-harboring *oqxAB* and ESBL- encoding genes embedded in agarose gel plugs was treated with S1 nuclease (TaKaRa, Dalian, China) and separated by PFGE alongside a standard lambda ladder PFG Marker (NEB, UK). Subsequently, Southern blot hybridization was performed with DNA probes speciﬁc for *oqxB*, *bla*_CTX-M-1G_ or *bla*_CTX-M-9G_, which were non-radioactively labeled with a DIG High Prime DNA labeling and detection kit (Roche Diagnostics, Mannheim, Germany). Plasmid DNA extraction was performed using a QIAGEN Plasmid Midi kit (QIAGEN, Germany). Plasmids of transconjugants were digested with the endonuclease *EcoR*I (TaKaRa Biotechnology, Dalian, China) to analyze the restriction fragment length polymorphism (RFLP) proﬁles.

### Molecular typing

To determine their genetic relatedness, chromosomal DNAs of 109 *E. coli* isolates randomly selected from the *oqxAB*-harboring isolates were digested with *Xba*I and subjected to pulsed-field gel electrophoresis (PFGE) according to a protocol described previously [30]. The DNA banding patterns were analysed using BioNumerics software version 2.5 (Applied Maths), and a cut-off value of 95% of the similarity values was chosen to indicate identical PFGE types. *Salmonella enterica* serotype Braenderup H9812 standards served as size markers.

## Results

### Antimicrobial susceptibility testing

Almost all the 696 clinical *E. coli* isolates in this study were highly resistant to nadidixic acid (96.1%), ampicillin (95.1%) and tetracycline (96.3%). The antimicrobial resistance rates to other antibiotics were as follows: chloramphenicol (85.3%), enrofloxacin (82.6%), doxycycline (79.6%), ciproﬂoxacin (76.3%), streptomycin (74.0%) levoﬂoxacin (73.6%), ﬂorfenicol (73.1%), gentamicin (64.4%) ceftiofur (46.3%), and amikacin (26.6%). Of the 696 isolates, 228 (32.8%) (97 from pigs, and 131 from avian) showed reduced susceptibility to cefotaxime (MIC≥2μg/mL), and all the 228 isolates were resistant to ceftiofur. ESBL production was detected by the screening method in 206 of the 228 isolates, representing 29.6% of the total 696 *E. coli* isolates.

### Prevalence of PMQR genes

Four hundred and twenty-nine (61.6%) of the 696 isolates were found to have at least one PMQR gene by PCR and sequencing of the PCR products. *oqxAB*, found in 328 isolates (47.1% of the total), was the most prevalent PMQR gene, followed by *qnrS* (14.5%) and *aac(6*’)*-Ib-cr* (14.4%). The number of isolates harboring *qnrB* and *qepA* was 48 (7.0%) and 21 (3.0%), respectively, but no isolate was positive for *qnrA*, *qnrC* or *qnrD*. In addition, 180 of the 328 *oqxAB*-positive isolates were detected to be linked with IS26. The combination types of PMQR genes in *E. coli* of different origin were listed in Table 1.

**Table 1:** Distribution of ESBL genes among 429 PMQR-positive *E. coli* isolates of food-producing animals.

PMQR genes (total number of isolates)	Origin (No. of isolates)	ESBL genes		No. of isolates producing ESBLs
		*bla*_CTX-M-9G_	*bla*_CTX-M-1G_	
*oqxAB* (207)	Avian (91)	*bla*_CTX-M-9G_		18
			*bla*_CTX-M-1G_	15
		*bla*_CTX-M-9G_	*bla*_CTX-M-1G_	2
	Swine (116)	*bla*_CTX-M-9G_		12
			*bla*_CTX-M-1G_	9
		*bla*_CTX-M-9G_	*bla*_CTX-M-1G_	2
*oqxAB*, *aac(6*’)*-Ib-cr* (39)	Avian (19)	*bla*_CTX-M-9G_		15
			*bla*_CTX-M-1G_	2
		*bla*_CTX-M-9G_	*bla*_CTX-M-1G_	1
	Swine (20)	*bla*_CTX-M-9G_		6
			*bla*_CTX-M-1G_	2
		*bla*_CTX-M-9G_	*bla*_CTX-M-1G_	1
*qnrS* (40)	Avian (17)	*bla*_CTX-M-9G_		3
			*bla*_CTX-M-1G_	4
	Swine (23)	*bla*_CTX-M-9G_		3
			*bla*_CTX-M-1G_	1
*oqxAB*, *qnrS* (32)	Avian (10)	*bla*_CTX-M-9G_		1
	Swine (22)	*bla*_CTX-M-9G_		7
			*bla*_CTX-M-1G_	1
*oqxAB*, *qnrS*, *aac(6*’)*-Ib-cr* (10)	Avian (4)	*bla*_CTX-M-9G_		1
	Swine (6)	*bla*_CTX-M-9G_		2
			*bla*_CTX-M-1G_	1
*qnrS*, *aac(6*’)*-Ib-cr* (3)	Swine (3)	*bla*_CTX-M-9G_		1
*oqxAB*, *qnrS*, *aac(6*’)*-Ib-cr*, *qnrB* (3)	Avian (3)	*bla*_CTX-M-9G_		2
			*bla*_CTX-M-1G_	1
*qnrS*, *qnrB* (3)	Avian (1)			0
	Swine (2)			0
*oqxAB*, *qnrS*, *qnrB* (2)	Avian (1)			0
	Swine (1)			0
*oqxAB*, *qnrS*, *qepA* (6)	Avian (1)			0
	Swine (5)	*bla*_CTX-M-9G_		4
*qnrS*, *qepA* (2)	Swine (2)			0
*oqxAB*, *qnrB* (15)	Avian (8)	*bla*_CTX-M-9G_		3
	Swine (7)	*bla*_CTX-M-9G_		5
			*bla*_CTX-M-1G_	1
*oqxAB*, *aac(6*’)*-Ib-cr*, *qnrB* (9)	Avian (8)	*bla*_CTX-M-9G_		6
			*bla*_CTX-M-1G_	1
	Swine (1)	*bla*_CTX-M-9G_		1
*aac(6*’)*-Ib-cr*, *qnrB* (3)	Avian (2)	*bla*_CTX-M-9G_		1
	Swine (1)	*bla*_CTX-M-9G_		1
*qnrB* (13)	Avian (7)		*bla*_CTX-M-1G_	1
	Swine (6)	*bla*_CTX-M-9G_		3
*oqxAB*, *qepA* (4)	Avian (1)	*bla*_CTX-M-9G_		1
	Swine (3)	*bla*_CTX-M-9G_		1
			*bla*_CTX-M-1G_	1
		*bla*_CTX-M-9G_	*bla*_CTX-M-1G_	1
*qepA* (6)	Avian (3)	*bla*_CTX-M-9G_		1
	Swine (3)	*bla*_CTX-M-9G_		1
			*bla*_CTX-M-1G_	1
*aac(6*’)*-Ib-cr*, *qepA* (2)	Swine (2)			0
*oqxAB*, *aac(6*’)*-Ib-cr*, *qepA* (1)	Avian (1)	*bla*_CTX-M-9G_		1
*aac(6*’)*-Ib-cr* (30)	Avian (16)	*bla*_CTX-M-9G_		2
			*bla*_CTX-M-1G_	4
	Swine (14)	*bla*_CTX-M-9G_		4
		*bla*_CTX-M-9G_	*bla*_CTX-M-1G_	1

### ESBL-encoding genes detection

ESBL-encoding genes were detected in most *E. coli* isolates with a cefotaxime MIC ≥ 2 μg/mL. CTX-M-type genes were found to be dominant in the isolates with ESBL production, and 191 isolates carried one or two CTX-M genes, representing 27.4% of the total 696 clinical *E. coli* isolates. *bla*_SHV_ and *bla*_TEM_ type ESBL- encoding genes were not found in any of these isolates. Among the 191 *bla*_CTX-M_-positive isolates, the number of isolates carrying *bla*_CTX-M-1G_ and *bla*_CTX-M-9G_ were 63 and 136, respectively, with 8 isolates carrying both *bla*_CTX-M-1G_ and *bla*_CTX-M-9G_ included. As the data of randomly sequenced *bla*_CTX-M_ that shown in Table 2, the most predominant CTX-M-encoding gene was *bla*_CTX-M-14_ (n=36), followed by *bla*_CTX-M-55_ (n=29). The most common CTX-M type in isolates from pigs was *bla*_CTX-M-14_, whereas *bla*_CTX-M-27_ was the most common type among isolates from avian (Table 2).

**Table 2:** Distribution of CTX-M subgroups and alleles amongst *Escherichia coli* isolates from different animal sources.

origin	ESBL(s) gene		No. of isolates
	*bla*_CTX-M-9_ Group	*bla*_CTX-M-1_ Group	
avian	*bla*_CTX-M-27_		21
avian	*bla*_CTX-M-14_		16
avian	*bla*_CTX-M-65_		5
avian	*bla*_CTX-M-125_		3
avian	*bla*_CTX-M-24_		1
avian	*bla*_CTX-M-14_	*bla*_CTX-M-55_	1
avian		*bla*_CTX-M-55_	18
avian		*bla*_CTX-M-15_	1
avian		*bla*_CTX-M-3_	1
swine	*bla*_CTX-M-14_		15
swine	*bla*_CTX-M-65_		11
swine	*bla*_CTX-M-27_		4
swine	*bla*_CTX-M-24_		3
swine	*bla*_CTX-M-125_		2
swine	*bla*_CTX-M-104_		2
swine	*bla*_CTX-M-90_		1
swine	*bla*_CTX-M-14_	*bla*_CTX-M-55_	4
swine		*bla*_CTX-M-55_	6
swine		*bla*_CTX-M-15_	1
swine		*bla*_CTX-M-3_	1

### *bla*_CTX-M_ genes among the PMQR-positive isolates

The distribution of ESBL-encoding genes among the 429 PMQR-positive *E. coli* isolates was shown in Table 1. One hundred and sixty of the 429 PMQR-positive isolates were detected to harbor CTX-M type genes, whereas, *bla*_CTX-M_ genes were detected in 31 of the 267 PMQR-negative isolates (P<0.001). As shown in Table 1, the detection of CTX-M type genes in isolates carrying *oqxAB* (127/328) was significantly higher than that in *oqxAB*-negative isolates (64/368) (P<0.001). For avian, the number of isolates carrying *oqxAB*-*bla*_CTX-M-9G_, *oqxAB*-*bla*_CTX-M-1G_, and *oqxAB*-*bla*_CTX-M-9G_-*bla*_CTX-M-1G_ were 48, 20, and 3, respectively. And there were 37, 15 and 4 isolates from pigs were found to harbor *oqxAB*-*bla*_CTX-M-9G_, *oqxAB*-*bla*_CTX-M-1G_, and *oqxAB*-*bla*_CTX-M-9G_-*bla*_CTX-M-1G_, respectively (Table 1).

### Molecular typing

Of the 109 isolates, 97 were successfully typed by PFGE, and a total of 88 different PFGE proﬁles were obtained, suggesting that most of the isolates in the study were from epidemiologically unrelated *E. coli* clones.

### Co-transferability of *oqxAB* and *bla*_CTX-M_ genes and plasmid analysis

**Figure 1:**
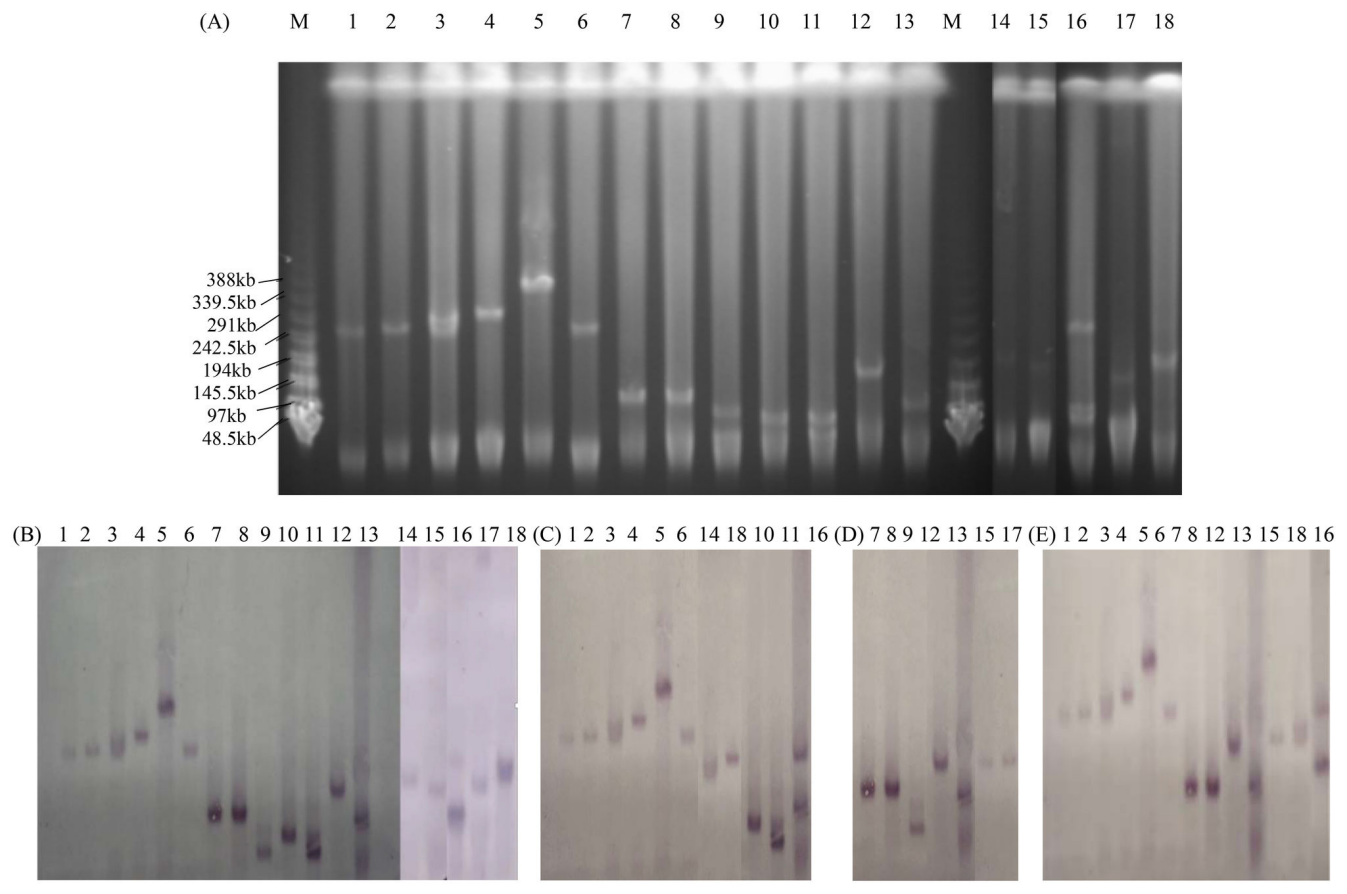
Plasmid analysis of transconjugants carrying *oqxAB* and *bla*_CTX-M_. (A) S1 nuclease-PFGE (B) Southern blot hybridization with the *oqxAB* probe. Lane 1-18: 45-6T, FS1Z4ST, a78T, CBJ3CT, a64T, 2YG4T, FS5E1DT, 42-2-2T, FS9Y1CT, A6T, 70zuT, FS3Z3GT, FS2Y1XT, FS6J2CT, 33-2T, 5weiT, FS3Z3CT, 7ganT; Lane M: lambda ladder PFG Marker. (C) Southern blot hybridization with the *bla*_CTX-M-9G_ probe (D) Southern blot hybridization with the *bla*_CTX-M-1G_ probe (E) Southern blot hybridization with the *floR* probe.

In this study, 84 transconjugants carrying *bla*_CTX-M_ were obtained from the 127 isolates with *oqxAB*-*bla*_CTX-M_. Eighteen of the 84 transconjugants were found to be also positive for *oqxAB*. Among the 18 transconjugants, 8 carried *oqxAB*, *bla*_CTX-M-9G_ and *aac(6*’)*-Ib-cr* simultaneously (Table 3). The result of S1 nuclease-PFGE shown in Figure 1A revealed that 14 transconjugants except FS3Z3GT, FS9Y1CT, 70zuT and 5weiT, carried only one plasmid (FS3Z3GT also harboring a very small plasmid not detected using S1-PFGE). As listed in Table 3, the IncFII (5 different alleles) replicon types were detected in 13 transconjugants, 7 of them carrying two replicons (FII in combination with FIB, HI2, or N). HI2 replicon type was found in 6 transconjugants, with 3 carrying other replicons. All transconjugants showed extremely high-level resistance to ampicillin, ceftiofur and cefotaxime, at the same level as the donor strains. For quinolones, the transconjugants showed 2~16-, and 4~16-fold increases in the MICs of nalidixic acid, and ciproﬂoxacin, respectively, when compared with the recipient C600. As shown in Table 3, all the transconjugants were multidrug-resistant and showed resistance to more than two non-β-lactam antimicrobial agents. Notably, 13 transconjugants were found to show high-level resistance to ﬂorfenicol, a veterinary antibiotic commonly used in veterinary medicine and aquaculture. The *floR* gene, which confers resistance to florfenicol, was found in all the 13 transconjugants. The results of Southern blot hybridization revealed that *oqxAB* and *bla*_CTX-M_ were located on the same plasmid in the all 18 transconjugants (Figure 1). And *floR* was also located on these plasmids in the 13 transconjugants resistant to florfenicol. Interestingly, isolates FS3Z3C and FS3Z3G sharing the same PFGE pattern were both resistant to flofenicol, however, the two F18: A-: B1 plasmids of their transconjugants were different (*floR* in FS3Z3GT, none in FS3Z3CT) (Table 3 and Figure 1E)

**Table 3:** Characteristics of the 18 *E. coli* transconjugants with plasmids harboring both *oqxAB* and *bla*_CTX-M-1G/9G_

Strain	Origin	Year	genes	MICs			Other resistance profiles	Plasmid replicon type	*EcoR*I PlasmidRFLP^a^
				NAL	CIP	CTX			
A6T	Duck	2007	*bla*_CTX-M-24_, *oqxAB aac(6*’)*-Ib-cr*	32	0.06	256	AMP,TET, CTI	F2:A-:B-	E
42-2T	Duck	2010	*bla*_CTX-M-55_, *oqxAB floR*	8	0.06	64	AMP, CHL, FFC, TET, CTI	F33:A-:B-	A1
FS5E1DT	Goose	2012	*bla*_CTX-M-55_, *oqxAB floR*	8	0.06	64	AMP, CHL, FFC, TET, CTI	F33:A-:B-	A1
FS6J2CT	Chicken	2012	*bla*_CTX-M-14_, *oqxAB*	64	0.125	16	AMP, CHL,TET, DOX, CTI	F14:A-:B-N	B
CBJ3CT	Chicken	2012	*bla*_CTX-M-14_, *oqxAB floR*	32	0.06	16	AMP, CHL, FFC, GEN, CTI	HI2, N	F3
FS1Z4ST	Pig	2012	*bla*_CTX-M-14_, *oqxAB aac(6*’)*-Ib-cr*, *floR*	32	0.125	8	AMP, CHL, FFC, TET GEN, KAN, CTI	HI2	F1
FS3Z3CT	Pig	2012	*bla*_CTX-M-55_, *oqxAB*	8	0.06	128	AMP, CHL,TET, DOX, CTI	F18:A-:B1	C
FS3Z3GT	Pig	2012	*bla*_CTX-M-55_, *oqxAB floR*	8	0.06	128	AMP, CHL, TET, DOX, FFC, GEN, CTI	F18:A-:B1	NA
A78T	Duck	2007	*bla*_CTX-M-27_, *oqxAB aac(6*’)*-Ib-cr*, *floR*	32	0.25	64	AMP, CHL, TET, FFC, GEN, AMK, CTI	HI2 F2:A-:B-	F4
7ganT	Pig	2010	*bla*_CTX-M-27_, *oqxAB aac(6*’)*-Ib-cr*, *floR*	64	0.25	64	AMP, CHL, TET, DOX, FFC, CTI	NT	G
70zuT	Pig	2010	*bla*_CTX-M-14_, *oqxAB*	16	0.125	16	AMP, CHL, GEN, CTI	F43:A-:B16	NA
5weiT	Pig	2010	*bla*_CTX-M-65_, *oqxAB aac(6*’)*-Ib-cr*, *floR*	64	0.25	16	AMP, CHL, TET, DOX, FFC, GEN, CTI	F2:A-:B-	NA
A64T	Duck	2007	*bla*_CTX-M-27_, *oqxAB floR, aac(6*’)*-Ib-cr*	32	0.25	32	AMP, CHL, TET, DOX, FFC, AMK, GEN, CTI	HI2 F2:A-:B-	F5
2YG4T	Duck	2011	*bla*_CTX-M-14_, *floR oqxAB*, *aac(6*’)*-Ib-cr*	64	0.25	2	AMP, CHL, TET, FFC, CTI, GEN	HI2	F2
FS9Y1CT	Duck	2012	*bla*_CTX-M-55_, *oqxAB*	64	0.25	32	AMP, CHL, DOX, TET, CTI	F33:A-:B-N	NA
FS2Y1XT	Duck	2012	*bla*_CTX-M-55_, *oqxAB*, *floR*	64	0.06	16	AMP, CHL, DOX, TET, FFC, CTI	F33:A-:B-	A2
33-2T	Duck	2010	*bla*_CTX-M-55_ *oqxAB*, *floR*	64	0.125	32	AMP, CHL, DOX, TET, FFC, GEN, CTI	F18:A-:B1	D
45-6T	Duck	2010	*bla*_CTX-M-14_ *aac(6*’)*-Ib-cr oqxAB*, *floR*	32	0.125	32	AMP, CHL, DOX, TET, FFC, GEN, CTI	HI2	F1
C600				4	0.015	0.125			

^a^ RFLP patterns differing by only a few bands (n=1 ~ 3) were assigned to the same RFLP profile. NA, not analysed.

AMP, ampicillin; CTX, cefotaxime; CTI, ceftiofur; AMK, amikacin; GEN, gentamicin; FFC, florfenicol; CHL, chloramphenicol; TET, tetracycline; DOX, doxycycline; NAL, nalidixic acid; CIP, ciprofloxacin;.

**Figure 2:**
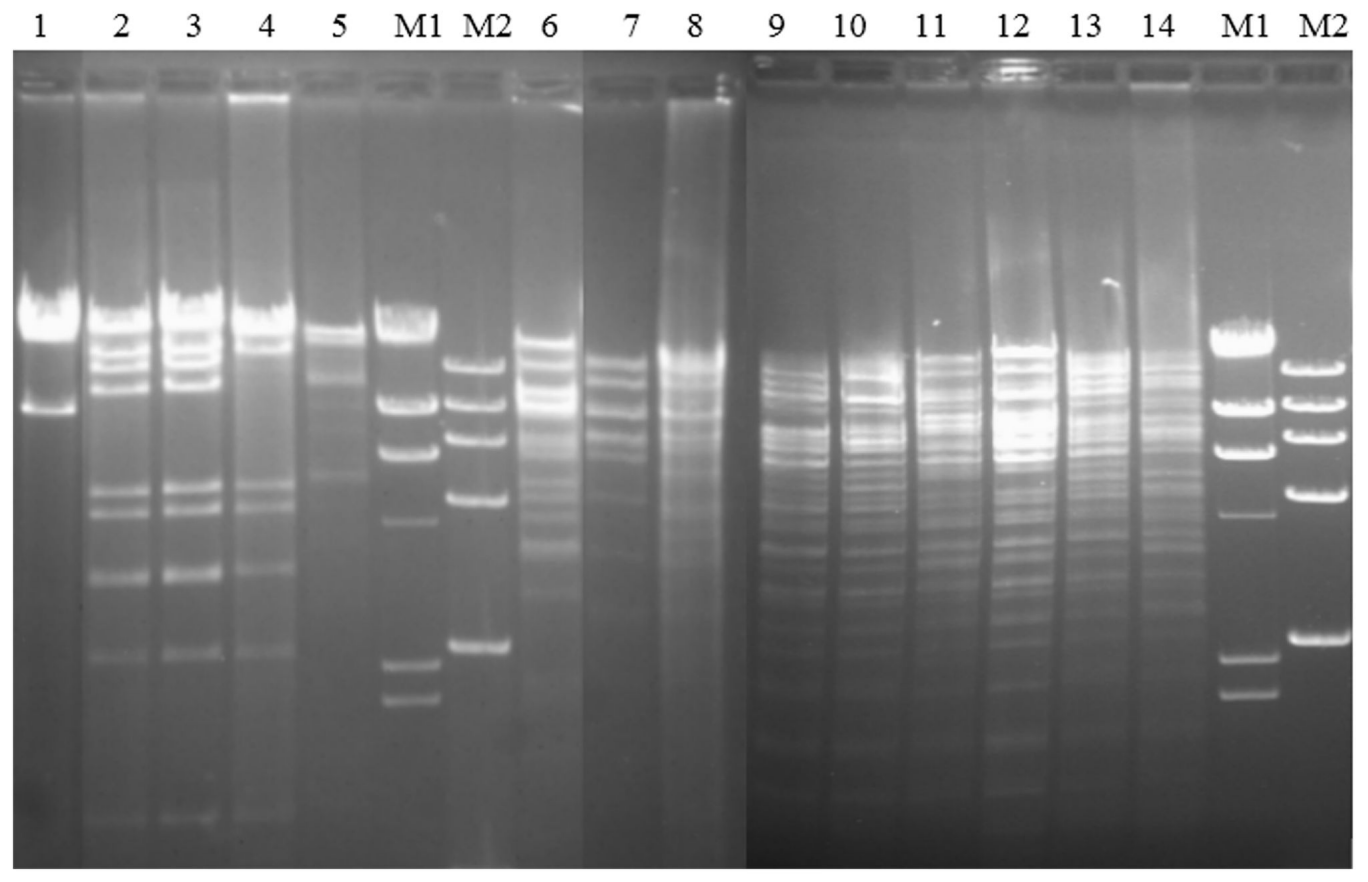
*EcoR*I restriction digestion proﬁles of plasmids co-harboring *oqxAB*-*bla*_CTX-M_ group genes from transconjugants containing only one plasmid. Lanes 1–14: A6T, 42-2T, FS5E1DT, FS2Y1XT, FS6J2CT, 7ganT, 33-2T, FS3Z3CT, 45-6T, 2YG4T, A64T, A78T, FS1Z4S, and CBJ3CT; Lane M1: λ-*Hind*III marker; Lane M2: DL15000.

As shown in Figure 2, the two F33:A-: B- plasmids p42-2 and pFS5E1DT shared the same *EcoR*I digestion profiles. This result could be confirmed by the positions of the bands in lane 7 and 8 in Figure 1. The *EcoR*I digestion proﬁle of plasmid from FS2Y1XT was only one band different from that of p42-2 and pFS5E1DT (Figure 2). As shown in Figure 2, the 6 HI2 plasmids carrying both *oqxAB* and *bla*_CTX-M-9G_ also showed the same or highly similar *EcoR*I digestion proﬁles. The plasmids from the other 5 transconjugants harboring only one plasmid, shared very different *EcoR*I digestion proﬁles.

## Discussion

In the present study, the prevalence of ESBLs in PMQR-positive (especially *oqxAB*) clinical *E. coli* isolates from food-producing animals in South China was investigated and the characteristics of plasmids carrying *oqxAB*-*bla*_CTX-M_ were also achieved. Surveys on the coexistence of *oqxAB* and ESBLs among Enterobacteriaceae have been reported [12,15,31], however, the characteristics of plasmids with *oqxAB*- *bla*_CTX-M_ were not analyzed in these previous studies. In the present study, a high prevalence (61.6%) of PMQR determinants was found in the 696 *E. coli* isolates from diseased food-producing animals, similar to our previous work [32]. The positive rate of *oqxAB* in *E. coli* from pigs in this study was similar to that found in *E. coli* from pigs in China [33]. Though olaquindox, the main substrate of efflux pump OqxAB, has been forbidden in poultry since 2000 due to its toxic side effects, the positive rate of *oqxAB* (46.2%) in *E. coli* from avian was significantly higher than that in *E. coli* isolates from chicken in 2002 in China [33]. This indicated the rapid dissemination of *oqxAB* in *E. coli* from animals in China in recent years. In this study, *oqxA* was found to be ﬂanked by IS26 in 54.9% of the *oqxAB*-carrying isolates, which suggests that the mobile element may play an important role in the dissemination of *oqxAB* among different *E. coli* strains. In this study, 322 isolates were resistant to ceftiofur and 70.8% of the ceftiofur-resistant isolates were found to be positive for ESBLs, which was similar to the result (68.3%) of a previous study [21]. There might be two reasons for the higher ceftiofur resistance rate: (i) there might be other β-lactamases not included in this study among these isolates, especially *bla*_CMY-2_, often conferring resistance to ceftiofur rather than cefotaxime; (ii) ceftiofur, one of the good substrates of AcrAB efflux pump [34], is often used to treat animal diseases and this long-term pressure will contribute to the presence of ceftiofur-resistant isolates. Among the 696 *E. coli* isolates studied, 29.6% of them were ESBL-producers and 27.4% carried CTX-M-β-lactamases, which were both much higher than the detection rates (13.1% and 12.4% respectively) in *E. coli* from healthy animals in a recent report [35] (P<0.001). The different incidence of ESBLs amongst *E. coli* isolates from food animals may be due to the use of third-generation cephalosporins in the diseased or dead food-producing animals in this study. At least ten types of *bla*_CTX-M_ genes were found in this study, indicating the *bla*_CTX-M_ genes in *E. coli* from food-producing animals in China were diverse. The predominant *bla*_CTX-M_ type in this study was *bla*_CTX-M-14_, similar result was also reported in *E. coli* isolates from humans and animals in China [35,36,37], however, *bla*_CTX-M-1_ was the most common type in some countries in Europe like England, and France [38,39].

We observed a significantly higher prevalence of CTX-M genes in the 429 PMQR-positive isolates (37.3%) than that (11.6%) in PMQR-negative isolates. This result supports previous findings that PMQR genes are often linked with ESBL production [9]. In addition, the detection rate of CTX-M type genes in *oqxAB*-positive isolates (38.7%) was significantly higher than that in *oqxAB*-negative isolates (17.4%) (P<0.001), indicating *bla*_CTX-M_ might have significant relationship with the new PMQR determinant, *oqxAB*. Among the 127 isolates with *oqxAB*-*bla*_CTX-M_, plasmids carrying *bla*_CTX-M_ from 84 isolates (66.1%) were conjugatively transferable, similar to the rate in a previous report in China [35]. Co-transferability of *oqxAB* and *bla*_CTX-M_ occurred in 18 (14.2%) of the 127 isolates, providing support for our previous hypothesis that *oqxAB* has correlation with ESBL. *oqxAB* and *bla*_CTX-M_ were confirmed to be located on the same plasmids in all the 18 isolates. To our knowledge, this is the first report of the prevalence of plasmids carrying *oqxAB* and *bla*_CTX-M_. Association of multiple antibiotic resistance genes on the same transferable plasmids has been an important mechanism of dissemination of multidrug resistance, and the transferable *oqxAB*-*bla*_CTX-M_ plasmids might explain in part the rapid increasing prevalence of *oqxAB* in *E. coli* of food-producing animals. Because OqxAB has a wide substrate specificity, the existence of its substrates in the environment will increase the resistances of *E. coli* isolates to fluoroquinolones and cephalosporins. In addition, *floR* was located on the plasmids carrying *oqxAB*-*bla*_CTX-M_ in 13 transconjugants, indicating that the application of florfenicol, commonly used in veterinary medicine and aquaculture, will also increase the resistances of *E coli* to fluoroquinolones and cephalosporins. In 8 transconjugants, *aac(6*’)*-Ib-cr* was also located on the plasmids carrying *oqxAB*-*bla*_CTX-M_, consistent with the findings that *aac(6*’)*-Ib-cr* is linked to *bla*_CTX-M_ in Enterobacteriaceae [9]. IncFII replicon types were detected in 13 of the 18 transconjugants, consistent with previous findings that most *bla*_CTX-M_ genes or *oqxAB* were found to be linked with IncFII plasmids [33,35]. In addition, HI2 plasmids were also often found to be linked with the co-dissemination of *oqxAB*- *bla*_CTX-M_ in this study. Three of the 6 HI2 plasmids were also positive for other replicon types (FII or N), and this might be explained by the presence of a multireplicon fusion of the HI2 plasmid with other replicon type plasmids, similar to a previous report [28]. Though the donors had different PFGE patterns, the F33:A-: B- and HI2 plasmids with *oqxAB*-*bla*_CTX-M_-*floR* genes shared highly similar RFLP profiles. This indicates that these two type plasmids might mediate the dissemination of *oqxAB*-*bla*_CTX-M_-*floR* genes in *E coli* from food-producing animals, and whether this represents global dissemination of these plasmids in the future is unclear and further work is required to clarify this.

In conclusion, we report a high prevalence (37.3%) of *bla*_CTX-M_ among PMQR-positive *E. coli* strains from diseased food-producing animals in China between 2002 and 2012. The co-dissemination of *oqxAB*, *floR* and *bla*_CTX-M_ genes in *E. coli* isolates from food-producing animals is mediated mainly by the F33:A-: B- and HI2 plasmids. These plasmids may promote the development of high-level multidrug-resistant isolates. Although a low prevalence (14.2%, 18 of the 127 *oqxAB*-*bla*_CTX-M_-positive isolates) of transferable plasmids carrying *oqxAB*-*bla*_CTX-M_ and dissemination of them mediated by similar plasmids were observed in the clinical *E. coli* isolates from food-producing animals, continued surveillance of the dissemination of these plasmids in Gram-negative bacteria is urgently needed because of the possibility that plasmids can be exchanged between bacteria from animals and those from humans. To our knowledge, this is the first report on the prevalence of the co-transferability of *oqxAB* and *bla*_CTX-M_ genes. This is also the first description of the co-existence of the *oqxAB*, *floR*, and *bla*_CTX-M_ on the same plasmid in *E. coli*.
